# No Evidence of Association Between Human 24-Hour Urinary Dopamine and Weight, BMI or Glucose Homeostasis in a Retrospective Analysis

**DOI:** 10.5812/ijem-166881

**Published:** 2026-01-31

**Authors:** Nele Mattelaer, Silke David, Nele Steenackers, Bart Van der Schueren, Nathalie Weltens, Roman Vangoitsenhoven

**Affiliations:** 1 Clinical and Experimental Endocrinology, Department of Chronic Diseases and Metabolism, KU Leuven, Leuven Belgium; 2 Laboratory for Brain-Gut Axis Studies, Translational Research in Gastrointestinal Disorders, Department of Chronic Diseases and Metabolism, KU Leuven, Leuven, Belgium; 3 Department of Endocrinology, University Hospitals Leuven, Leuven, Belgium

**Keywords:** Obesity, Dopamine, Glucose

## Abstract

**Background:**

Dopamine is an essential neurotransmitter involved in motivation and reward. In obesity, altered dopaminergic responses to food may drive excess energy intake, implicating dopamine in the pathophysiology of obesity. Additionally, dopamine may act as an anti-incretin, potentially facilitating the development of type 2 diabetes.

**Objectives:**

This retrospective study investigated associations between urinary dopamine, weight, Body Mass Index (BMI), plasma glucose, and hemoglobin A1c (HbA1c).

**Methods:**

Twenty-four-hour urinary dopamine concentrations measured with liquid chromatography tandem mass spectrometry between January 2000 and October 2020 were retrieved from the University Hospitals Leuven clinical database. Records with dopamine disruption disorders or medications, psychiatric conditions, prior bariatric surgery, active malignancies, diabetes mellitus other than type 2 diabetes, chronic renal insufficiency, or urinary creatinine concentration outside the reference range were excluded, yielding 178 records. Spearman correlations and multiple linear regression models were performed; statistical significance was set at P < 0.05.

**Results:**

Twenty-four-hour urinary dopamine concentrations positively correlated with body weight and negatively with HbA1c, but these correlations became non-significant when corrected for age, sex, and estimated glomerular filtration rate (eGFR). The only remaining significant correlation was between age and twenty-four-hour urinary dopamine (β = -1.2, P = 0.030).

**Conclusions:**

No significant correlations were found between twenty-four-hour urinary dopamine and weight, BMI, or glucose homeostasis, potentially due to the absence of dietary control. As dopamine measurements came from a clinical database, generalizability is limited. Prospective dedicated studies are needed to disentangle the role of peripheral dopamine in the pathophysiology of obesity.

## 1. Background

Obesity is a multifactorial disease that has been linked to altered eating behaviour (1). Food intake regulation involves an interplay between homeostatic and hedonic circuitries at the central level, mediated by bidirectional gut-brain signals (1). An imbalance between these control mechanisms might lead to altered food reward responses, which could then promote the observed altered eating behaviour in obesity (2).

Dopamine, a catecholamine that is produced from its precursor L-DOPA, is peripherally mainly produced in the intestines and kidneys (3), while the substantia nigra, hypothalamus, and ventral tegmental area are major central production sites (4). Dopamine is essential in food reward processing, and many research efforts have aimed to link dopaminergic alterations to obesity, but findings are inconsistent (1, 5). Further, dopamine has also been proposed to act as an ‘anti-incretin’, making it a potential mediating factor in type 2 diabetes (T2D) (6). Postprandially, dopamine is released from the foregut almost simultaneously with glucagon-like peptide 1 and glucose-dependent insulinotropic polypeptide, which reinforce postprandial insulin secretion (6). Dopamine has also been found to suppress beta-cell proliferation in vitro (6, 7), and some in vitro and (limited) in vivo studies showed that dopamine diminishes insulin secretion (6, 8-11). Chronically elevated dopamine levels might therefore facilitate T2D development by diminishing insulin secretion and sensitivity (12).

## 2. Objectives

It has been proposed that dopamine can cross the blood-brain barrier, but it remains unclear whether human peripheral dopamine concentrations are related to body composition and glucose homeostasis (13-15). Since urinary dopamine levels reflect the daily production of dopamine (15), we aimed to study available urinary dopamine measurements and their relations with weight, Body Mass Index (BMI), and measures of glucose homeostasis.

## 3. Methods

### 3.1. Study Design

In this retrospective study, urinary dopamine measurements performed between January 2000 and October 2020 were retrieved from the results server of the clinical laboratory of the University Hospitals Leuven. Hydrochloric acid was added to the urine collection to prevent the degradation of catecholamines (16). Dopamine concentration was determined using liquid chromatography tandem mass spectrometry (LC-MS/MS). The electronic patient records were queried for demographic and clinical data at the timing of sample collection. The database was then screened to exclude cases of possible disruptors of dopamine signaling (including movement and thyroid disorders, prolactinomas, paragangliomas, pheochromocytomas, anti-Parkinson's medication, ADHD medication, anti-nausea medication, antipsychotics, anti-prolactinomas, selective serotonin and noradrenalin reuptake inhibitors, tricyclic antidepressants), psychiatric conditions, prior bariatric surgery, active malignancies, diabetes mellitus other than T2D, chronic renal insufficiency ≥ type 3b, urinary creatinine concentration outside the reference range (women: 0.72 - 1.51 g/24h; men: 0.98 - 2.20 g/24h). The study was approved by the ethical committee of the University Hospitals Leuven (OBC-MP016270) and was conducted in accordance with the Declaration of Helsinki.

### 3.2. Statistical Analysis

Statistical analyses were performed using R studio 1.4.1106. Data are expressed as median (IQR) or as absolute and relative frequencies for qualitative variables. Kruskal-Wallis tests were used to assess differences in quantitative variables among BMI groups, and categorical variables were compared using Chi-square tests. Wilcoxon rank-sum tests were used to assess differences in quantitative variables among sex groups. Correlations between variables were analyzed using Spearman correlation coefficients. Multiple linear regression models were employed to estimate the relationship between 24-hour urinary dopamine concentrations (the gold standard of dopamine excretion) and independent variables. Multicollinearity was checked. Statistical significance was set at P < 0.05.

## 4. Results

### 4.1. Population Characteristics

Between 2000 and 2020, 5878 urinary dopamine measurements were performed. Of those, 636 records had simultaneous measurements of weight, glucose, and hemoglobin A1c (HbA1c). Considering the above-mentioned exclusion criteria, 178 records were included in the final analysis. The most common clinical indication for urinary dopamine testing was as part of extensive routine testing for hypertension in an academic hospital; other indications included incidentaloma/lesions of the adrenal gland or suspicions of pheochromocytoma, excessive (night) sweating, or malaise. Fifty-seven people had a BMI < 25, sixty-one had a BMI between 25-29.9, and sixty people had a BMI ≥ 30 (Table 1). The proportion of people with prediabetes or T2D was highest among people with obesity (P = 0.014). Cardiovascular risk markers were significantly different across BMI groups, including glucose (P = 0.025), HbA1c (P = 0.0021), triglycerides (P = 0.001), high-density lipoprotein (HDL) (P = 3.68e-07), and calculated low-density lipoprotein (LDL) (P = 0.048).

**Table 1.** Clinical Characteristics of The Study Population ^a, b^

Variables	Total Group		BMI < 25		BMI 25 - 29.9		BMI ≥ 30		P - Value
	n	Values	n	Values	n	Values	n	Values	
**Sex **	178								0.095
Male		83 (46.6)		20 (35.1)		35 (57.4)		28 (46.7)	
Female		95 (53.4)		37 (64.9)		26 (42.6)		32 (53.3)	
**Age (y)**	178	55 (45 - 64)	57	53 (43 - 64)	61	56 (46 - 64)	60	56 (45.8 - 65.5)	0.35
**Weight (kg)**	178	81.25 (68.1 - 92.9)	57	62.1 (57.0 - 70.1)	61	81.1 (73.8 - 88.1)	60	98.55 (89.35 - 107.15)	4.54e-25 ^c^
**BMI (kg/m** ^**2**^ **)**	178	27.8 (24.4 - 31.2)	57	22.8 (21.6 - 23.9)	61	27.7 (26.0 - 29.0)	60	33.1 (31.2 - 37.0)	
**Diabetes **	178		57		61		60		6.07e-34 ^c^
Prediabetes		56 (31.5)		16 (28.1)		20 (32.8)		20 (33.3)	0.014 ^c^
T2D		43 (24.2)		7 (12.3)		14 (23.0)		22 (36.7)	
**Nicotine use**	174		55		61		58		0.98
No		151 (86.7)		48 (87.3)		53 (86.9)		50 (86.2)	
Yes		23 (13.2)		7 (12.7)		8 (13.1)		8 (13.8)	
**Biochemical**									
Glucose (mg/dL)	178	100 (92 - 125.5)	57	97 (86 - 113)	61	102 (93 - 119)	60	103 (94.75 - 138.5)	0.025 ^c^
HbA1c (%)	178	5.7 (5.3 - 6.2)	57	5.5 (5.2 - 5.8)	61	5.7 (5.3 - 6.0)	60	5.9 (5.6 - 6.85)	0.0021 ^c^
Creatinine plasma (mg/dL)	178	0.91 (0.77 - 1.04)	57	0.83 (0.71 - 1.03)	61	0.93 (0.81 - 1.06)	60	0.93 (0.82 - 1.00)	0.11
Creatinine urine (ug/24h)	178	1.21 (1.00 - 1.48)	57	1.08 (0.85 - 1.36)	61	1.28 (1.11 - 1.55)	60	1.26 (1.00 - 1.53)	0.014 ^c^
eGFR (mL/min/1.73m^2^)	116	0.93 (0.25)	40	0.89 (0.29)	42	0.95 (0.24)	34	0.93 (0.23)	0.6183
Cholesterol (mg/dL)	149	197 (161 - 231)	44	195 (169 - 216.25)	52	194 (159.25 - 223.75)	53	205 (159.25 - 223.75)	0.48
Triglycerides (mg/dL)	149	117 (92 - 177)	44	101.5 (72.25 - 133.25)	52	132.5 (97.75 - 209.50)	53	126 (107 - 189)	0.001 ^c^
HDL (mg/dL)	148	51 (39.75 - 64)	44	64.5 (54.75 - 73.25)	51	48 (38 - 58)	53	48 (35 - 53)	3.68e-07 ^c^
Calculated LDL (mg/dL)	147	110 (85.5 - 144)	44	102 (81.5 - 132)	50	97.5 (75.75 - 143.5)	53	121 (95 - 155)	0.048 ^c^
**Blood pressure**	178								
Systolic blood pressure (mmHg)		153 (131.3 - 180.0)	57	151 (125 - 180)	61	150 (130 - 176)	60	157 (130 - 176)	0.27
Diastolic blood pressure (mmHg)		89 (78 - 100)	57	86 (73 - 98)	61	86 (79 - 100)	60	89 (78 - 100)	0.50
**Antihypertensive drug use**	178		57		61		60		0.20
0		45 (25.3)		24 (42.1)		12 (19.7)		9 (15.0)	
1		45 (25.3)		14 (24.6)		17 (27.9)		14 (23.3)	
2		42 (23.6)		11 (19.3)		16 (26.2)		15 (25.0)	
> 2		46 (25.8)		8 (14.0)		16 (26.2)		22 (36.7)	
**Antidepressant use**	178		57		61		60		0.81
No		161 (90.4)		52 (91.2)		56 (91.8)		53 (88.3)	
Yes		17 (9.6)		5 (8.8)		5 (8.2)		7 (11.7)	
**Antidiabetic drug use**	178		57		61		60		0.20
0		149 (83.7)		53 (93.0)		52 (85.2)		44 (73.3)	
1		20 (11.2)		3 (5.3)		6 (9.8)		11 (18.3)	
2		9 (5.1)		1 (1.8)		3 (4.9)		5 (8.3)	
**Contraceptive drug use**			37		26		32		0.51
No		79 (83.2)		29 (78.4)		21 (80.8)		29 (90.6)	
Yes		16 (16.8)		8 (21.6)		5 (19.2)		3 (9.4)	

^a^ Values are presented as No. (%) unless otherwise indicated.

^b^ Kruskal-Wallis tests were used to assess differences in quantitative variables between groups. Chi-square tests were used to assess differences in categorical variables between groups. Results are presented as median (interquartile range) or as absolute frequency (relative frequency).

^c^ P < 0.05.

### 4.2. Comparison of Urinary Dopamine Concentration Across Body Mass Index Groups

Urinary dopamine concentration (μg/L) and daily urinary dopamine excretion (μg/24h) were compared between body mass index (BMI) groups. No statistically significant difference was found in urinary dopamine concentration (μg/L: BMI < 25: 106 (69.5-175); BMI 25 - 29.9: 116.7 (84 - 188.7); BMI ≥ 30: 122.1 (88.65 - 202.85); P = 0.42) nor total daily dopamine excretion (μg/24h: BMI < 25: 181 (147.8 - 249.6); BMI 25 - 29.9: 205 (166.2 - 252.7); BMI ≥ 30: 207.25 (156.8 - 243.45); P = 0.64).

### 4.3. Relationship Between Urinary Dopamine Concentration, Weight, Body Mass Index, and Glucose Homeostasis

**Figure 1.**
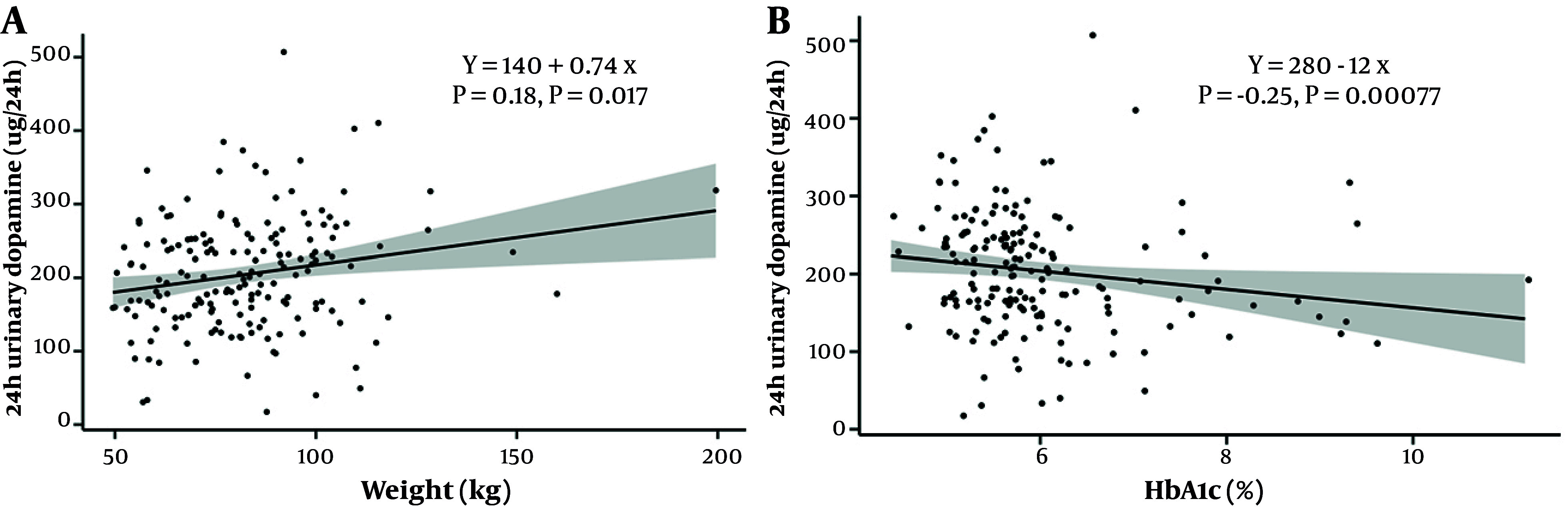
A, Positive correlation between dopamine excretion and weight; B, negative correlation between dopamine excretion and hemoglobin A1c. ρ = Spearman correlation. Significant correlation: P < 0.05

We explored potential correlations between 24-hour urinary dopamine concentration and weight, BMI, and measures of glucose homeostasis (hemoglobin A1c (HbA1c) and glucose). A significant positive correlation was found between weight and 24-hour urinary dopamine (r = 0.178, P = 0.017) (Figure 1), but not between BMI and 24-hour urinary dopamine (r = 0.055, P = 0.47). HbA1c showed a significant negative correlation with 24-hour urinary dopamine concentration (r = -0.250, P = 0.00077) (Figure 1), but plasma glucose did not (r = -0.139, P = 0.065). However, in a multiple linear regression model, weight and HbA1c lost their significant correlation with 24-hour urinary dopamine concentration. In this model, predictor variables were selected based on their significant correlation with 24-hour urinary dopamine concentrations (weight, HbA1c, estimated glomerular filtration rate (eGFR), age, sex). Age was the only predictor variable that remained significant (β = -1.2, P = 0.030). Supplementary Table S1 provides the variance inflation factors of each predictor variable in this model, and Supplementary Figure S1 in Supplementary File provides the residual plot. To assess the robustness of findings, we reran the model without the diabetes population, which did not affect the results (Supplementary File). Similarly, exclusion of nicotine users, prediabetes, or use of antidiabetic drugs did not affect the results (Table S2 in Supplementary File). When creatinine-adjusted urinary dopamine concentrations were used instead of 24-hour urinary dopamine concentration, sex emerged as a significant predictor, but none of the variables of interest were significant (Supplementary File).

## 5. Discussion

Dopamine is essential in attributing reward value to food (1). Several studies have investigated central dopamine signaling in the context of obesity and peripheral dopamine in the context of type 2 diabetes (T2D), mostly in vitro (5, 6). In this study, we investigated the potential relation between peripheral dopamine, specifically 24-hour urinary dopamine concentrations, and weight, Body Mass Index (BMI), plasma glucose concentration, and hemoglobin A1c (HbA1c).

There was a significant positive correlation between 24-hour dopamine excretion and body weight, implying that more body mass is associated with more dopamine production, but when corrected for age, sex, HbA1c, and estimated glomerular filtration rate (eGFR), this was no longer significant. Moreover, when weight was corrected for height (i.e., BMI), no significant correlation was found. This contrasts with a study by Basolo et al., which reported a positive Pearson correlation between BMI and 24-hour urinary dopamine concentration (13). In their analysis, data from two studies were pooled to study the relation between dopamine and ad libitum energy intake. Only non-smokers who used no medication were included.

In our study, data were retrieved from a clinical database, where there was a primary indication for dopamine measurements. Underlying clinical differences, or differences in smoking and medication use, might therefore explain the opposing outcomes. In addition, different analytical techniques (high performance liquid chromatography vs liquid chromatography tandem mass spectrometry (LC-MS/MS)) and statistical approaches (Pearson correlation vs Spearman correlation and multiple linear regression) were used, which could lead to further discrepancies in the significance of the findings. Basolo et al. excluded people with T2D, although neither study found a significant correlation between urinary dopamine and plasma glucose. Further, exclusion of records with T2D did not affect the results of the current study (Supplementary File).

Lastly, Basolo et al. excluded people over the age of 55. Since age was a negative predictor of 24-hour dopamine excretion in the current analysis, differences in age range might also explain some of the differences in results between the two studies. Importantly, neither study was able to account for diet, although this has been proposed to influence urinary dopamine levels (17). To date, no dedicated prospective studies exist that focus on peripheral dopamine levels in relation to body composition or metabolic health.

Nonetheless, dopamine has long been a target for anti-obesity pharmacotherapy. Naltrexone-bupropion relies on the synergistic mechanism of action of an opioid antagonist and a noradrenalin-dopamine reuptake inhibitor to diminish food craving (18), causing significant weight loss compared to placebo (19). A recent study found that in obesity, striatal dopamine release after intragastric lipid infusion is impaired and not restored after diet-induced weight loss (20). In animal studies, however, it has been shown that a Western diet high in calories induces central changes in dopamine signaling (13). This illustrates the need to better understand the role of dopamine in human obesity pathophysiology and its central-peripheral dynamics to create more targeted weight loss strategies.

In our study, a significant negative correlation was found between 24-hour urinary dopamine and HbA1c, but not with plasma glucose. After controlling for age, sex, weight, and eGFR, the correlation with HbA1c was no longer significant. Similarly, Basolo et al. found no significant correlation between 24-hour urinary dopamine and plasma glucose concentration, controlled for age, sex, race, and body fat (13). An important caveat is, however, that neither study was designed for dopamine evaluation. As mentioned, dietary information was not available, although it very likely interferes with urinary dopamine measurement (17). The absence of a statistically significant relation between HbA1c, glucose, BMI, and weight should therefore be interpreted with caution. Given the observed variability, it remains possible that a true association exists but was not detected due to limited statistical power. The only statistically significant result that we found is a negative correlation with age. It has been shown that dopamine-producing neurons decrease with aging, and it is therefore likely that this has introduced heterogeneity in our study cohort (21). Similarly, several studies have suggested a higher striatal dopaminergic tone in women than in men (22).

So far, evidence on sex differences in peripheral dopamine is lacking, but the inclusion of both men and women might as well have introduced some variability. In addition, while it is evident from several studies that nicotine use affects central dopamine signaling, little is known about the effects on peripheral dopamine (23). Yet, in the current study, we found no significant effect of the exclusion of nicotine users on the adjusted results.

It is currently unclear how well 24-hour urinary dopamine concentrations reflect systemic dopamine production. As mentioned, urinary dopamine is the most commonly used measure of peripheral dopamine, whereas circulating dopamine is not often measured, especially not simultaneously with urinary levels. In this study, the aim was to explore potential relationships between urinary dopamine and anthropometric measurements. As a next step, it would be valuable to conduct dedicated prospective studies to simultaneously assess systemic and urinary dopamine levels within the same population. Particularly, studies conducted in people with obesity and following weight loss could provide valuable insights. For example, by investigating people who underwent bariatric surgery, intra-individual changes in dopamine and body composition could be compared. Additionally, this could help disentangle the potential role of dopamine as an anti-incretin, as proposed by some in view of the foregut hypothesis. This hypothesis states that by bypassing the proximal part of the intestines (a major production site of circulating dopamine), the secretion of gastrointestinal factors that promote insulin resistance will decrease (6). Diet, and particularly protein content, is a known influencer of 24-hour urinary dopamine concentrations (17). Because of the retrospective nature of this study, we did not have this information. Future studies should include detailed dietary assessment to further explore the link between diet and peripheral dopamine concentrations, body composition, and glucose homeostasis. In addition, measuring central and peripheral levels of dopamine together would help clarify how peripheral concentrations reflect central signaling. It has been proposed that dopamine can cross the blood-brain barrier, though limited studies have studied the relation between peripheral and central levels (15).

This study has several limitations. First, a retrospective study cannot infer causality. Next, dopamine excretion was measured in clinical care in a workup for several potential conditions, possibly introducing bias in the study population, despite strict exclusion criteria. In addition, because of the retrospective nature of the study, no information on the duration of diabetes or prediabetes was available. Finally, no information on diet or eating behaviour was available, which may mediate the potential link between body composition and dopamine (24).

In conclusion, in this retrospective analysis consisting of 178 records in which a 24-hour urine analysis was performed, we found no significant correlation between urinary dopamine concentrations and weight, BMI, or measures of glucose homeostasis when controlling for age, sex, and eGFR.

## supplementary material

ijem-24-1-166881-s001.pdf

## Data Availability

The dataset presented in the study is available on request from the corresponding author during submission or after publication.
