# Supplementary material for: No Evidence of Association Between Human 24-Hour Urinary Dopamine and Weight, BMI or Glucose Homeostasis in a Retrospective Analysis
Source: Int J Endocrinol Metab. 2026 Jan 31;24(1):e166881. doi: 10.5812/ijem-166881 (PMC13187684; doi:10.5812/ijem-166881)
Supplement: ijem-24-1-166881-s001.pdf [file ijem-24-1-166881-s001.pdf]

Supplementary File

Supplementary Table S1. Variance inflation factors of each predictor variable in the multiple regression model of 24-hour urinary dopamine concentration

Predictor variable	VIF
Weight	1.513088
HbA1c	1.195528
Age	1.481952
Sex	1.425395
eGFR	1.397614

Supplementary Figure S1. Residual plot of the multiple regression model of 24-hour urinary dopamine concentration showing the distribution of residuals around fitted values

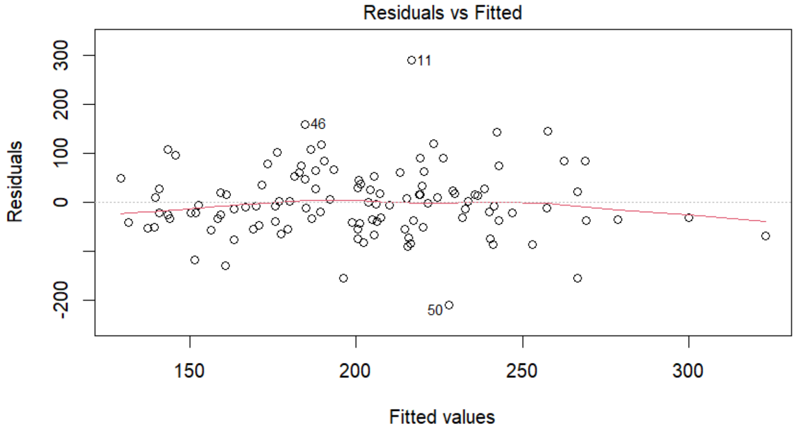

Supplementary Table S2. Multiple linear regression models of 24-hour urinary dopamine

	**24-hour urinary dopamine (µg/24h) Total group**	**24-hour urinary dopamine (µg/24h) No T2D**	**24-hour urinary dopamine (µg/24h) No T2D and prediabetes**	**24-hour urinary dopamine (µg/24h) No antidiabetics**	**24-hour urinary dopamine (µg/24h) No nicotine users**
Multiple R-Squared	n = 178<br>R^2=0.2278<br>f^2 = 0.2950013<br>p = 2.522e-05	n = 135<br>R^2 = 0.1869<br>f^2 = 0.229861<br>p = 0.002175	n = 79<br>R^2 = 0.1882<br>f^2 = 0.2318305<br>p = 0.07236	n = 149<br>R^2 = 0.1781<br>f^2 = 0.216693<br>p = 0.001986	n = 151<br>R^2 = 0.2532<br>f^2 = 0.3390466<br>p = 6.519e-05
Intercept	218.4320 ± 75.4349<br>Partial R^2 = 0.07082584<br>f^2 =0.0762245<br>p = 0.00457	263.4683 ± 125.8264<br>Partial R^2 = 0.0469503290<br>f^2 = 0.04926326<br>p = 0.03911	223.53537 ± 263.26955<br>Partial R^2 = 0.0151071438<br>f^2 = 0.01533887<br>p = 0.400	159.5832 ± 105.3068<br>Partial R^2 = 0.023602855<br>f^2 = 0.02417342<br>p = 0.1330	223.7425 ± 83.1093<br>Partial R^2 = 0.074527891<br>f^2 = 0.08052959<br>p = 0.00847
Weight	0.65 ± 0.47<br>Partial R^2= 0.01734094<br>f^2 = 0.01764695<br>p = 0.16635	0.4361 ± 0.5454<br>Partial R^2 = 0.0071340315<br>f^2 = 0.007185292<br>p = 0.42602	0.13389 ± 0.74375<br>Partial R^2 = 0.0006890421<br>f^2 = 0.0006895172<br>p = 0.858	0.3372 ± 0.5222<br>Partial R^2 = 0.004368688<br>f^2 = 0.004387857<br>p = 0.5201	0.5000 ± 0.5268<br>Partial R^2 = 0.009911544<br>f^2 = 0.01001077<br>p = 0.34506
HbA1c	-9.7156 ± 8.1286<br>Partial R^2 = 0.01282065<br>f^2 = 0.01298715	-3.6890 ± 20.0462<br>Partial R^2 = 0.0003803689<br>f^2 = 0.0003805136	16.99232 ± 45.70952<br>Partial R^2 = 0.0029316960<br>f^2 = 0.002940316	8.2112 ± 16.1141<br>Partial R^2 = 0.002725759<br>f^2 = 0.002733209	-8.6064 ± 8.5656<br>Partial R^2 = 0.011092843<br>f^2 = 0.01121727

	p = 0.23457	p = 0.85441	p = 0.712	p = 0.6115	p = 0.31770
Age	-1.2017 ± 0.5457<br>Partial R^2 = 0.04222228<br>f^2 = 0.04408359<br>p = 0.02975	-1.5533 ± 0.5820<br>Partial R^2 = 0.0741021405<br>f^2 = 0.08003274<br>p = 0.00904	-2.10598 ± 0.83409<br>Partial R^2 = 0.1194378114<br>f^2 = 0.1356381<br>p = 0.015	-1.2632 ± 0.5984<br>Partial R^2 = 0.044800825<br>f^2 = 0.04690208<br>p = 0.0374	-1.2688 ± 0.6090<br>Partial R^2 = 0.046006353<br>f^2 = 0.04822501<br>p = 0.04006
Sex	-22.1455 ± 15.9491<br>Partial R^2 = 0.01722517<br>f^2 = 0.01752708<br>p = 0.16779	-22.5799 ± 17.5000<br>Partial R^2 = 0.0183623881<br>f^2 = 0.01870587<br>p = 0.20030	-23.33119 ± 24.02555<br>Partial R^2 = 0.0196698853<br>f^2 = 0.02006455<br>p = 0.336	-29.9419 ± 18.1377<br>Partial R^2 = 0.027886194<br>f^2 = 0.02868614<br>p = 0.1021	-33.0737 ± 18.1030<br>Partial R^2 = 0.035760524<br>f^2 = 0.03708677<br>p = 0.07102
eGFR	0.7983 ± 0.4281<br>Partial R^2 = 0.03063628<br>f^2 = 0.03160453<br>p = 0.06491	0.2804 ± 0.4718<br>Partial R^2= 0.0039530526<br>f^2 = 0.003968741<br>p = 0.55380	0.05944 ± 0.65984<br>Partial R^2 = 0.0001726159<br>f^2 = 0.0001726457<br>p = 0.929	0.7044 ± 0.4762<br>Partial R^2 = 0.022510248<br>f^2 = 0.02302863<br>p = 0.1424	0.9280 ± 0.4804<br>Partial R^2 = 0.039802821<br>f^2 = 0.04145276<br>p = 0.05657

ANALYSIS OF 24-HOUR URINARY DOPAMINE IN A NON-DIABETIC POPULATION-

Population characteristics

135 records were included in the final analysis. 50 people had a BMI <25, 47 had a BMI between 25-29.9 and 38 people had a BMI ≥ 30. Triglycerides (p=0.029) and HDL (p<0.0001) were significantly different across BMI groups.

Comparison of urinary dopamine concentration across BMI groups

Urinary dopamine concentration (μg/l) and daily urinary dopamine excretion (μg/24h) were compared between BMI groups. No statistically significant difference was found in urinary dopamine concentration (μg/L: BMI<25: 109 (69.93-198.63); BMI 25-29.9: 129.5 (86.05-192.45); BMI ≥ 30: 127.6 (94.33-219.35); p = 0.48) nor total daily dopamine excretion (μg/24h: BMI<25: 203.85 (157.58-252.28); BMI 25-29.9: 230.1 (168.85; 258.9); BMI ≥ 30: 215.1 (98.5-149.5); p=0.56).

Relationship between urinary dopamine concentration, weight, BMI and glucose homeostasis

We explored potential correlations between 24-hour urinary dopamine concentration and weight, BMI and measures of glucose homeostasis (HbA1c and glucose). A significant positive correlation was found between weight and 24-hour urinary dopamine (r=0.175, p=0.043), but not between BMI and 24-hour urinary dopamine (r=0.051, p=0.56). HbA1c showed a significant negative correlation with 24-hour urinary dopamine

concentration (r=-0.209, p=0.015), but plasma glucose did not (r=-0.097, p=0.26). However, in a multiple linear regression model, with weight, Hb1Ac, eGFR, age and sex as predictor variables, weight and HbA1C lost their significant correlation with 24-hour urinary dopamine concentration. Age was the only predictor variable that remained significant (β= -1.55, p=0.0090).

ANALYSIS OF CREATININE-ADJUSTED URINARY DOPAMINE CONCENTRATION

Comparison of creatinine-adjusted urinary dopamine concentration (μg/g) across BMI groups

Creatinine-adjusted urinary dopamine concentration was compared between BMI groups. No statistically significant difference was found (BMI<25: 172.76 (126.67-218.19); BMI 25-29.9: 159.73 (126.94-193.77); BMI ≥ 30: 172.76 (121.23-185.81) (p=0.44).

Relationship between creatinine-adjusted urinary dopamine concentration, weight, BMI and glucose homeostasis

We explored potential correlations between creatinine-adjusted urinary dopamine concentration and weight, BMI and measures of glucose homeostasis (HbA1c and glucose). A significant negative correlation was found between creatinine-adjusted urinary dopamine concentration and weight (r=-0.20, p=0.0072). Yet, in a multiple linear regression model, weight lost its significant correlation with creatinine-adjusted urinary dopamine concentration. In this model, predictor variables were selected based on their significant correlation with creatinine-adjusted urinary dopamine concentration (weight, age, sex). Age (β= -0.80, p=0.0025) and sex (β= 39.19, p= 0.0000034) were significant predictor variables in this

Commented [NM1]: I didn't include eGFR in this model since we already use creatinine-adjusted dopamine

Commented [NM2R1]: Of zou ik eGFR toch toevoegen aangezien de reviewer zegt: 'Urinary dopamine can be influenced by renal function beyond eGFR'. Als ik eGFR toevoeg is eGFR een significante predictor

model. BMI (r=-0.11, p=0.15), plasma glucose (r=-0.0049, p=0.95) and HbA1c (r=-0.12, p=0.098) did not have a significant correlation with creatinine-adjusted urinary dopamine concentration.

ANALYSIS OF 3-METHOXYTYRAMINE (3-MT)

Comparison of urinary 3-methoxytyramine (3-MT) concentration across BMI groups

Of the 178 records, urinary 3-MT concentration (µg/l) and daily urinary 3-MT excretion (µg/24h) was available in only 86 and 31 records, respectively. These concentrations were compared between BMI groups. Urinary 3-MT concentration was significantly different between the overweight and obesity group (µg/l: BMI<25: 83 (57.5-105.5); BMI 25-29.9: 112 (80.5-149); BMI ≥ 30: 110 (76-129.25); p = 0.030). Daily urinary 3-MT excretion was not significantly different between groups (µg/24h: BMI<25: 126 (97.5-149.5); BMI 25-29.9 (181 (140.5-193); BMI ≥ 30: 147 (103-198)).

Relationship between urinary 3-MT concentration, weight, BMI and glucose homeostasis

We explored potential correlations between 24-hour 3-MT concentration and weight, BMI and measures of glucose homeostasis (HbA1c and glucose). A significant positive correlation was found between weight and 24-hour 3-MT (r=0.45, p=0.011), but not 24-hour 3-MT and BMI (r=0.21, p=0.26), HbA1c (r=0.29, p=0.12) or glucose (r=0.31, p=0.091). Multiple linear regression was performed, with predictor variables selected

based on their significant correlation with 24-hour urinary 3-MT concentrations (weight, 24-hour urinary creatinine excretion, sex). In this model, weight was no longer significantly associated with 24-hour 3-MT excretion.
